# Supplementary material for: Exploring the Potential of Olfactory Receptor Circulating RNA Measurement for Preeclampsia Prediction and Its Linkage to Mild Gestational Hypothyroidism
Source: Int J Mol Sci. 2023 Nov 24;24(23):16681. doi: 10.3390/ijms242316681 (PMC10706743; doi:10.3390/ijms242316681)
Supplement: Supplementary file 1 [file ijms-24-16681-s001.zip › ijms-2623467-supplementary.pdf]

SUPPLEMENTARY MATERIAL

Supplementary Table S1. Olfactory Receptors (ORs) differentially expressed genes (DEG) list. We show the logarithmic fold change (logFC) of the 320 ORs from mild nontreated gestational hypothyroidism (GHT) compared with the healthy thyroid pregnancy (HTP). We also show the 317 ORs differentially expressed in preeclampsia (PEC) and 320 ORs in HTP. Healthy nonpregnant women libraries were used to evaluate the DEG in HTP.

Symbol	LogFC.GHT_HTP	LogFC.PEC_HTP	LogFC.HTP_NPG	Gene name
OR10A2	-3,22E+13	-2,78E+14	2,63E+14	olfactory receptor family 10 subfamily A member 2
OR10A3	-4,32E+14	-3,58E+14	2,90E+14	olfactory receptor family 10 subfamily A member 3
OR10A4	-3,34E+14	-2,07E+14	2,94E+14	olfactory receptor family 10 subfamily A member 4
OR10A5	-3,30E+14	-2,49E+14	2,30E+14	olfactory receptor family 10 subfamily A member 5
OR10A6	-6,19E+14	-2,37E+14	2,76E+14	olfactory receptor family 10 subfamily A member 6 (gene/pseudogene)
OR10A7	-3,57E+14	-3,13E+14	3,09E+14	olfactory receptor family 10 subfamily A member 7
OR10AG1	-2,54E+13	-6,44E+14	2,98E+14	olfactory receptor family 10 subfamily AG member 1
OR10C1	-3,56E+14	-3,60E+11	3,20E+14	olfactory receptor family 10 subfamily C member 1 (gene/pseudogene)
OR10G3	-3,44E+14	-3,83E+14	2,00E+14	olfactory receptor family 10 subfamily G member 3
OR10G4	-3,18E+14	-3,53E+14	2,96E+14	olfactory receptor family 10 subfamily G member 4
OR10G7	-3,51E+14	-4,45E+13	2,37E+14	olfactory receptor family 10 subfamily G member 7
OR10G8	-3,61E+14	-3,26E+14	3,15E+14	olfactory receptor family 10 subfamily G member 8
OR10G9	-4,97E+14	-3,72E+14	3,45E+14	olfactory receptor family 10 subfamily G member 9
OR10H1	-3,37E+14	-5,54E+12	2,55E+14	olfactory receptor family 10 subfamily H member 1
OR10H2	-2,51E+14	-3,83E+14	2,21E+14	olfactory receptor family 10 subfamily H member 2
OR10H3	-4,48E+14	-2,72E+14	2,99E+14	olfactory receptor family 10 subfamily H member 3
OR10H4	-3,88E+14	-3,81E+14	2,68E+14	olfactory receptor family 10 subfamily H member 4
OR10H5	-2,56E+14	-3,26E+14	2,46E+12	olfactory receptor family 10 subfamily H member 5
OR10J1	-2,76E+14	-4,70E+14	3,13E+14	olfactory receptor family 10 subfamily J member 1
OR10J3	-2,32E+14	-2,75E+14	2,42E+14	olfactory receptor family 10 subfamily J member 3
OR10J5	-3,97E+14	-3,62E+14	2,68E+14	olfactory receptor family 10 subfamily J member 5
OR10K1	-2,68E+14	-2,76E+14	2,91E+14	olfactory receptor family 10 subfamily K member 1
OR10K2	-2,81E+14	-2,71E+14	2,26E+14	olfactory receptor family 10 subfamily K member 2

OR10P1	-6,60E+14	-3,17E+14	2,36E+14	olfactory receptor family 10 subfamily P member 1
OR10Q1	-3,87E+14	-1,81E+13	3,33E+13	olfactory receptor family 10 subfamily Q member 1
OR10R2	-4,61E+14	-3,28E+14	2,41E+14	olfactory receptor family 10 subfamily R member 2
OR10S1	-2,43E+14	-3,85E+14	1,84E+13	olfactory receptor family 10 subfamily S member 1
OR10T2	-2,85E+14	-4,16E+14	2,71E+14	olfactory receptor family 10 subfamily T member 2
OR10V1	-2,45E+14	-2,67E+14	2,80E+14	olfactory receptor family 10 subfamily V member 1
OR10W1	-2,82E+14	-3,08E+14	2,58E+14	olfactory receptor family 10 subfamily W member 1
OR10X1	-7,31E+14	-2,88E+13	2,81E+14	olfactory receptor family 10 subfamily X member 1 (gene/pseudogene)
OR10Z1	-2,25E+14	-2,43E+14	2,50E+13	olfactory receptor family 10 subfamily Z member 1
OR11G2	-3,23E+14	-2,53E+14	3,02E+14	olfactory receptor family 11 subfamily G member 2
OR11H1	-2,82E+13	-3,11E+14	3,13E+14	olfactory receptor family 11 subfamily H member 1
OR11H12	-3,93E+14	-4,02E+14	3,25E+14	olfactory receptor family 11 subfamily H member 12
OR11H2	-3,09E+14	-2,76E+14	3,47E+14	olfactory receptor family 11 subfamily H member 2
OR11H4	-3,33E+14	-1,81E+14	1,83E+14	olfactory receptor family 11 subfamily H member 4
OR11H6	-2,13E+14	-3,90E+14	2,95E+14	olfactory receptor family 11 subfamily H member 6
OR11L1	-3,43E+14	-5,97E+14	1,96E+14	olfactory receptor family 11 subfamily L member 1
OR12D2	-3,80E+14	-3,36E+14	2,29E+14	olfactory receptor family 12 subfamily D member 2 (gene/pseudogene)
OR12D3	-4,48E+14	-5,25E+14	3,01E+14	olfactory receptor family 12 subfamily D member 3
OR13C2	-3,72E+14	-4,29E+14	2,54E+14	olfactory receptor family 13 subfamily C member 2
OR13C3	-3,22E+14	-1,75E+14	2,47E+14	olfactory receptor family 13 subfamily C member 3
OR13C4	-2,78E+14	-3,89E+14	2,82E+14	olfactory receptor family 13 subfamily C member 4
OR13C5	-4,87E+13	-2,93E+14	2,15E+14	olfactory receptor family 13 subfamily C member 5
OR13C8	-3,63E+14	-2,94E+14	2,04E+14	olfactory receptor family 13 subfamily C member 8
OR13C9	-4,09E+14	-2,99E+14	3,25E+14	olfactory receptor family 13 subfamily C member 9
OR13F1	-3,12E+14	-3,53E+14	2,43E+14	olfactory receptor family 13 subfamily F member 1
OR13G1	-1,18E+14	-5,08E+14		olfactory receptor family 13 subfamily G member 1
OR13H1	-2,40E+14	-3,56E+14	2,94E+14	olfactory receptor family 13 subfamily H member 1
OR13J1	-2,47E+14	-3,33E+14	2,22E+14	olfactory receptor family 13 subfamily J member 1

OR14A16	-2,89E+14	-2,97E+14	2,72E+14	olfactory receptor family 14 subfamily A member 16
OR14C36	-4,33E+14	-3,08E+14	3,32E+14	olfactory receptor family 14 subfamily C member 36
OR14I1	-1,93E+13	-3,76E+14	2,40E+14	olfactory receptor family 14 subfamily I member 1
OR14J1	-2,31E+14	-2,77E+14	3,07E+14	olfactory receptor family 14 subfamily J member 1
OR1A1	-4,46E+14	-2,88E+14	2,54E+14	olfactory receptor family 1 subfamily A member 1 (gene/pseudogene)
OR1A2	-3,48E+14	-2,53E+14	3,48E+14	olfactory receptor family 1 subfamily A member 2
OR1B1	-7,48E+13	-2,94E+14	3,42E+14	olfactory receptor family 1 subfamily B member 1 (gene/pseudogene)
OR1D2	-4,87E+13	-3,60E+14	3,15E+14	olfactory receptor family 1 subfamily D member 2
OR1D5	-2,86E+14	-3,24E+13	2,03E+14	olfactory receptor family 1 subfamily D member 5
OR1E1	-2,65E+14	-3,47E+14	3,08E+14	olfactory receptor family 1 subfamily E member 1
OR1E2	-3,12E+14	-2,90E+14	2,67E+12	olfactory receptor family 1 subfamily E member 2
OR1F1	-3,50E+14	-2,76E+14	1,96E+13	olfactory receptor family 1 subfamily F member 1
OR1G1	-2,23E+14	-4,49E+14	3,72E+14	olfactory receptor family 1 subfamily G member 1
OR1I1	-4,01E+14	-2,25E+14	2,89E+14	olfactory receptor family 1 subfamily I member 1
OR1J1	-3,01E+14	-2,99E+14	2,00E+13	olfactory receptor family 1 subfamily J member 1
OR1J2	-3,77E+14	-3,67E+14	2,26E+14	olfactory receptor family 1 subfamily J member 2
OR1J4	-3,70E+14	-3,89E+14	1,92E+14	olfactory receptor family 1 subfamily J member 4
OR1L1	-2,90E+14	-2,77E+14	2,31E+14	olfactory receptor family 1 subfamily L member 1
OR1L3	-5,14E+14	-3,08E+14	3,06E+14	olfactory receptor family 1 subfamily L member 3
OR1L4	-1,93E+14	-1,46E+14	1,75E+14	olfactory receptor family 1 subfamily L member 4
OR1L6	-2,03E+14	-1,99E+14		olfactory receptor family 1 subfamily L member 6
OR1L8	-2,36E+13	-3,69E+14	3,05E+14	olfactory receptor family 1 subfamily L member 8
OR1M1	-2,43E+14	-3,72E+14	2,59E+14	olfactory receptor family 1 subfamily M member 1
OR1N2	-3,24E+14	-2,41E+14	2,22E+14	olfactory receptor family 1 subfamily N member 2
OR1Q1	-3,88E+14	-2,82E+13		olfactory receptor family 1 subfamily Q member 1
OR1S1	-2,60E+14	-2,77E+14	2,99E+14	olfactory receptor family 1 subfamily S member 1 (gene/pseudogene)
OR1S2	-3,44E+14	-4,25E+14	3,55E+14	olfactory receptor family 1 subfamily S member 2
OR2A12	-3,65E+14	-2,32E+14	2,75E+14	olfactory receptor family 2 subfamily A member 12

OR2A14	-4,00E+14	-3,34E+14	2,00E+14	olfactory receptor family 2 subfamily A member 14
OR2A2	-3,43E+14	-2,84E+14	3,25E+14	olfactory receptor family 2 subfamily A member 2
OR2A25	-3,33E+14	-2,34E+14	2,72E+14	olfactory receptor family 2 subfamily A member 25
OR2A5	-7,43E+14	-2,77E+13	3,67E+14	olfactory receptor family 2 subfamily A member 5
OR2AG1	-3,60E+14	-3,19E+14	2,19E+14	olfactory receptor family 2 subfamily AG member 1 (gene/pseudogene)
OR2AG2	-4,23E+14	-4,15E+14	2,35E+14	olfactory receptor family 2 subfamily AG member 2
OR2AT4	-5,06E+14	-2,94E+14	3,13E+14	olfactory receptor family 2 subfamily AT member 4
OR2B2	-2,11E+14	-2,75E+14	1,78E+14	olfactory receptor family 2 subfamily B member 2
OR2B3	-4,13E+14	-3,09E+14	2,98E+14	olfactory receptor family 2 subfamily B member 3
OR2B6	-1,01E+14	-2,78E+14		olfactory receptor family 2 subfamily B member 6
OR2D2	-4,68E+14	-3,19E+13	2,41E+14	olfactory receptor family 2 subfamily D member 2
OR2D3	-2,75E+14	-2,83E+14	2,12E+13	olfactory receptor family 2 subfamily D member 3
OR2F1	-2,00E+14	-4,31E+14	2,03E+14	olfactory receptor family 2 subfamily F member 1 (gene/pseudogene)
OR2F2	-4,11E+14	-3,48E+14	1,65E+14	olfactory receptor family 2 subfamily F member 2
OR2G2	-3,02E+14	-3,79E+14	2,17E+13	olfactory receptor family 2 subfamily G member 2
OR2G3	-4,75E+14	-3,24E+14	2,37E+14	olfactory receptor family 2 subfamily G member 3
OR2G6	-3,69E+14	-3,36E+14	1,82E+14	olfactory receptor family 2 subfamily G member 6
OR2H2	-2,29E+14	-2,05E+14	1,96E+14	olfactory receptor family 2 subfamily H member 2
OR2J3	-3,18E+14	-2,64E+14	2,43E+14	olfactory receptor family 2 subfamily J member 3
OR2K2	-3,44E+14	-4,37E+13	2,77E+14	olfactory receptor family 2 subfamily K member 2
OR2L3	-2,94E+14	-2,84E+14	2,47E+14	olfactory receptor family 2 subfamily L member 3
OR2M1P	-3,93E+14	-2,07E+14	3,44E+14	olfactory receptor family 2 subfamily M member 1 pseudogene
OR2M2	-2,13E+14	-2,57E+13	1,84E+14	olfactory receptor family 2 subfamily M member 2
OR2M3	-3,28E+14	-2,36E+14		olfactory receptor family 2 subfamily M member 3
OR2M4	-2,45E+14	-3,08E+14	3,87E+14	olfactory receptor family 2 subfamily M member 4
OR2M5	-3,28E+14	-4,46E+14	4,13E+14	olfactory receptor family 2 subfamily M member 5
OR2M7	-1,56E+14	-2,69E+14		olfactory receptor family 2 subfamily M member 7

OR2S2	-2,58E+14	-5,03E+14	2,76E+14	olfactory receptor family 2 subfamily S member 2 (gene/pseudogene)
OR2T1	-3,32E+14	-3,51E+14	2,90E+14	olfactory receptor family 2 subfamily T member 1
OR2T10	-2,54E+14	-2,56E+14	1,58E+14	olfactory receptor family 2 subfamily T member 10
OR2T12	-4,11E+14		2,67E+14	olfactory receptor family 2 subfamily T member 12
OR2T2	-2,50E+13	-2,93E+14	2,40E+14	olfactory receptor family 2 subfamily T member 2
OR2T27	-3,67E+14	-3,76E+13	3,20E+14	olfactory receptor family 2 subfamily T member 27
OR2T29	-2,87E+14	-2,46E+14	3,45E+14	olfactory receptor family 2 subfamily T member 29
OR2T35	-2,27E+14	-1,94E+13	1,88E+14	olfactory receptor family 2 subfamily T member 35
OR2T4	-3,51E+14	-4,06E+14	3,15E+14	olfactory receptor family 2 subfamily T member 4
OR2T5	-6,14E+14	-1,74E+13	2,31E+14	olfactory receptor family 2 subfamily T member 5
OR2T6	-2,82E+14	-3,64E+14	1,91E+14	olfactory receptor family 2 subfamily T member 6
OR2V2	-3,71E+14	-3,17E+14	2,58E+14	olfactory receptor family 2 subfamily V member 2
OR2W1	-5,75E+14	-1,71E+14	2,36E+14	olfactory receptor family 2 subfamily W member 1
OR2W3	-1,89E+14		1,40E+14	olfactory receptor family 2 subfamily W member 3
OR2W5	-2,24E+14	-3,58E+14	2,81E+14	olfactory receptor family 2 subfamily W member 5 (gene/pseudogene)
OR2Y1	-2,61E+14	-2,65E+14	1,60E+14	olfactory receptor family 2 subfamily Y member 1
OR2Z1	-3,41E+14	-3,31E+14	2,12E+14	olfactory receptor family 2 subfamily Z member 1
OR3A1	-3,17E+14	-2,43E+14	1,57E+14	olfactory receptor family 3 subfamily A member 1 (gene/pseudogene)
OR3A3	-3,56E+14	-5,70E+14	3,05E+14	olfactory receptor family 3 subfamily A member 3
OR3A4P	-2,73E+14	-3,60E+14	3,09E+14	olfactory receptor family 3 subfamily A member 4 pseudogene
OR4A15	-3,06E+14	-3,44E+14	1,90E+14	olfactory receptor family 4 subfamily A member 15
OR4A16	-2,69E+14	-3,21E+14	2,58E+14	olfactory receptor family 4 subfamily A member 16
OR4A47	-2,68E+14	-2,85E+14	2,36E+14	olfactory receptor family 4 subfamily A member 47
OR4A5	-3,33E+14	-4,09E+14	4,53E+14	olfactory receptor family 4 subfamily A member 5
OR4B1	-3,36E+14	-3,62E+14	2,53E+14	olfactory receptor family 4 subfamily B member 1
OR4C12	-6,28E+14	-6,28E+14	2,35E+14	olfactory receptor family 4 subfamily C member 12
OR4C13	-2,71E+14	-3,01E+14	3,65E+14	olfactory receptor family 4 subfamily C member 13

OR4C15	-4,69E+14	-5,41E+14	4,46E+14	olfactory receptor family 4 subfamily C member 15
OR4C16	-4,17E+14	-2,92E+14	3,49E+13	olfactory receptor family 4 subfamily C member 16 (gene/pseudogene)
OR4C3	-4,17E+14	-3,62E+14	2,07E+14	olfactory receptor family 4 subfamily C member 3
OR4C46	-3,18E+14	-3,39E+13	3,00E+14	olfactory receptor family 4 subfamily C member 46
OR4C6	-3,74E+14	-3,12E+14	3,55E+14	olfactory receptor family 4 subfamily C member 6
OR4D10	-3,29E+14	-2,81E+14	1,96E+14	olfactory receptor family 4 subfamily D member 10
OR4D11	-4,63E+14	-3,30E+14	2,81E+14	olfactory receptor family 4 subfamily D member 11
OR4D2	-3,20E+14	-3,08E+14	1,88E+14	olfactory receptor family 4 subfamily D member 2
OR4D5	-2,89E+14	-3,27E+14	2,51E+13	olfactory receptor family 4 subfamily D member 5
OR4D6	-2,55E+14	-4,53E+14	1,94E+14	olfactory receptor family 4 subfamily D member 6
OR4D9	-3,79E+14	-3,20E+14	2,69E+14	olfactory receptor family 4 subfamily D member 9
OR4E2	-3,44E+14	-3,05E+13	2,64E+14	olfactory receptor family 4 subfamily E member 2 (gene/pseudogene)
OR4F15	-3,11E+14	-2,77E+14	1,65E+14	olfactory receptor family 4 subfamily F member 15
OR4F16	-2,32E+14	-1,03E+14	1,40E+14	olfactory receptor family 4 subfamily F member 16
OR4F17	-3,12E+14	-1,88E+14	3,11E+14	olfactory receptor family 4 subfamily F member 17
OR4F21	-2,14E+14	-1,27E+14	1,83E+14	olfactory receptor family 4 subfamily F member 21
OR4F29	-3,60E+14	-1,62E+14	2,16E+14	olfactory receptor family 4 subfamily F member 29
OR4F3	-3,15E+14	-1,03E+14	1,69E+14	olfactory receptor family 4 subfamily F member 3
OR4F4	-2,74E+14	-1,91E+14	3,42E+14	olfactory receptor family 4 subfamily F member 4
OR4F5	-3,85E+14	-1,19E+14	2,79E+14	olfactory receptor family 4 subfamily F member 5
OR4F6	-4,25E+14	-2,69E+13	2,53E+14	olfactory receptor family 4 subfamily F member 6
OR4K1	-3,54E+14	-2,49E+14	3,48E+14	olfactory receptor family 4 subfamily K member 1
OR4K13	-4,12E+14	-5,70E+14	2,14E+14	olfactory receptor family 4 subfamily K member 13
OR4K14	-5,27E+14	-3,17E+14	2,36E+14	olfactory receptor family 4 subfamily K member 14
OR4K15	-4,23E+14	-3,35E+14	2,97E+14	olfactory receptor family 4 subfamily K member 15
OR4K17	-2,96E+14	-5,45E+14		olfactory receptor family 4 subfamily K member 17
OR4K2	-3,53E+14	-4,78E+14	2,93E+14	olfactory receptor family 4 subfamily K member 2
OR4K5	-3,07E+13	-3,00E+14	2,88E+14	olfactory receptor family 4 subfamily K member 5

OR4L1	-3,52E+13	-3,17E+14	4,05E+14	olfactory receptor family 4 subfamily L member 1 (gene/pseudogene)
OR4M1	-4,59E+14	-4,89E+14	3,37E+14	olfactory receptor family 4 subfamily M member 1
OR4M2	-3,89E+14	-4,34E+14	3,63E+14	olfactory receptor family 4 subfamily M member 2
OR4N2	-3,08E+14	-4,32E+14	3,09E+14	olfactory receptor family 4 subfamily N member 2
OR4N3P	-4,21E+14	-4,30E+14	2,92E+14	olfactory receptor family 4 subfamily N member 3 pseudogene
OR4N4	-2,85E+14	-2,50E+14	2,36E+14	olfactory receptor family 4 subfamily N member 4
OR4N5	-3,85E+14	-3,47E+14	2,86E+14	olfactory receptor family 4 subfamily N member 5
OR4Q3	-3,57E+13	-1,73E+14	2,72E+14	olfactory receptor family 4 subfamily Q member 3
OR4S1	-3,94E+14	-5,23E+13	3,06E+14	olfactory receptor family 4 subfamily S member 1
OR4X1	-3,46E+14	-3,60E+14	3,51E+14	olfactory receptor family 4 subfamily X member 1 (gene/pseudogene)
OR4X2	-2,91E+14	-2,65E+14	3,46E+14	olfactory receptor family 4 subfamily X member 2 (gene/pseudogene)
OR51A2	-2,34E+14	-4,42E+14	2,02E+14	olfactory receptor family 51 subfamily A member 2
OR51A4	-2,77E+14	-3,34E+14	2,69E+13	olfactory receptor family 51 subfamily A member 4
OR51A7	-3,87E+14	-3,88E+14	3,51E+14	olfactory receptor family 51 subfamily A member 7
OR51B2	-2,47E+14	-2,24E+14	3,13E+14	olfactory receptor family 51 subfamily B member 2 (gene/pseudogene)
OR51B4	-3,77E+14	-3,22E+14	2,85E+14	olfactory receptor family 51 subfamily B member 4
OR51B6	-3,59E+14	-3,42E+14	2,57E+14	olfactory receptor family 51 subfamily B member 6
OR51D1	-3,88E+14	-2,55E+12	2,73E+14	olfactory receptor family 51 subfamily D member 1
OR51F1	-3,41E+14	-4,71E+14	2,73E+14	olfactory receptor family 51 subfamily F member 1 (gene/pseudogene)
OR51F2	-1,89E+13	-3,88E+14	2,52E+14	olfactory receptor family 51 subfamily F member 2
OR51G1	-2,51E+14	-2,35E+14	1,84E+14	olfactory receptor family 51 subfamily G member 1 (gene/pseudogene)
OR51G2	-5,10E+14	-4,98E+13	3,04E+13	olfactory receptor family 51 subfamily G member 2
OR51I1	-2,34E+14	-3,18E+14	3,05E+14	olfactory receptor family 51 subfamily I member 1
OR51I2	-4,25E+14	-2,35E+14	2,90E+13	olfactory receptor family 51 subfamily I member 2
OR51L1	-2,86E+13	-3,16E+14	2,79E+14	olfactory receptor family 51 subfamily L member 1
OR51M1	-2,28E+14	-2,35E+14	2,01E+14	olfactory receptor family 51 subfamily M member 1

OR51Q1	-3,44E+14	-2,57E+14	2,17E+14	olfactory receptor family 51 subfamily Q member 1 (gene/pseudogene)
OR51S1	-3,45E+14	-4,70E+14	3,51E+14	olfactory receptor family 51 subfamily S member 1
OR51T1	-2,57E+14	-3,27E+14	2,96E+14	olfactory receptor family 51 subfamily T member 1
OR51V1	-3,07E+13	-3,97E+14	3,02E+14	olfactory receptor family 51 subfamily V member 1
OR52A1	-2,14E+14	-3,17E+14	2,58E+14	olfactory receptor family 52 subfamily A member 1
OR52A5	-2,71E+14	-3,59E+14	3,08E+14	olfactory receptor family 52 subfamily A member 5
OR52B2	-2,69E+14	-1,25E+14	2,47E+14	olfactory receptor family 52 subfamily B member 2
OR52B4	-2,67E+11	-2,74E+14	3,06E+14	olfactory receptor family 52 subfamily B member 4 (gene/pseudogene)
OR52D1	-2,89E+14	-3,74E+14	2,67E+14	olfactory receptor family 52 subfamily D member 1 (gene/pseudogene)
OR52E2	-3,63E+14	-3,09E+14	2,74E+14	olfactory receptor family 52 subfamily E member 2
OR52E4	-2,48E+14	-2,87E+14	4,85E+14	olfactory receptor family 52 subfamily E member 4
OR52E6	-2,65E+13	-4,08E+14	2,17E+14	olfactory receptor family 52 subfamily E member 6
OR52E8	-4,82E+13	-3,87E+14	2,91E+13	olfactory receptor family 52 subfamily E member 8
OR52I2	-1,75E+14	-2,64E+14	1,56E+14	olfactory receptor family 52 subfamily I member 2
OR52J3	-2,89E+14	-3,19E+14	2,51E+14	olfactory receptor family 52 subfamily J member 3
OR52L1	-2,24E+14	-2,11E+14	2,40E+14	olfactory receptor family 52 subfamily L member 1 (gene/pseudogene)
OR52M1	-4,58E+14	-3,33E+14	2,26E+14	olfactory receptor family 52 subfamily M member 1
OR52N1	-6,92E+14	-3,48E+14	3,18E+14	olfactory receptor family 52 subfamily N member 1
OR52N2	-2,07E+13	-3,57E+14	2,39E+14	olfactory receptor family 52 subfamily N member 2
OR52N5	-2,73E+14	-2,59E+14	2,12E+14	olfactory receptor family 52 subfamily N member 5
OR52R1	-3,45E+14	-2,84E+13	3,14E+14	olfactory receptor family 52 subfamily R member 1 (gene/pseudogene)
OR56A1	-4,05E+13	-3,01E+14	2,67E+14	olfactory receptor family 56 subfamily A member 1
OR56A3	-3,60E+14	-3,65E+14	2,54E+14	olfactory receptor family 56 subfamily A member 3
OR56A5	-3,33E+14	-3,25E+14	2,45E+14	olfactory receptor family 56 subfamily A member 5
OR56B4	-2,78E+14	-1,79E+14	2,49E+14	olfactory receptor family 56 subfamily B member 4
OR5A2	-3,15E+14	-4,22E+14	2,88E+14	olfactory receptor family 5 subfamily A member 2
OR5AK2	-6,27E+14	-4,46E+14	3,50E+14	olfactory receptor family 5 subfamily AK member 2

OR5AK4P	-4,39E+14	-4,27E+14	3,31E+14	olfactory receptor family 5 subfamily AK member 4 pseudogene
OR5AN1	-2,83E+14	-2,87E+14	2,96E+14	olfactory receptor family 5 subfamily AN member 1
OR5AP2	-1,90E+14	-3,76E+14	4,20E+14	olfactory receptor family 5 subfamily AP member 2
OR5AR1	-4,45E+14	-2,93E+14	2,17E+14	olfactory receptor family 5 subfamily AR member 1 (gene/pseudogene)
OR5AS1	-2,80E+14	-1,84E+13	2,05E+14	olfactory receptor family 5 subfamily AS member 1
OR5AU1	-2,70E+14	-3,41E+13	2,88E+14	olfactory receptor family 5 subfamily AU member 1
OR5B12	-3,12E+13	-3,42E+13	3,24E+13	olfactory receptor family 5 subfamily B member 12
OR5B17	-1,82E+14	-3,29E+14	2,84E+14	olfactory receptor family 5 subfamily B member 17
OR5B2	-3,28E+11	-2,36E+14	2,49E+14	olfactory receptor family 5 subfamily B member 2
OR5B3	-2,42E+14	-3,24E+14		olfactory receptor family 5 subfamily B member 3
OR5C1	-2,73E+14	-1,81E+14	1,83E+14	olfactory receptor family 5 subfamily C member 1
OR5D13	-2,49E+14	-3,03E+14	1,93E+14	olfactory receptor family 5 subfamily D member 13 (gene/pseudogene)
OR5D14	-3,57E+14	-2,83E+14	2,84E+14	olfactory receptor family 5 subfamily D member 14
OR5D16	-6,81E+14	-5,00E+14	4,01E+14	olfactory receptor family 5 subfamily D member 16
OR5D18	-7,35E+14	-4,22E+14	3,44E+14	olfactory receptor family 5 subfamily D member 18
OR5E1P	-5,99E+14	-5,99E+14	2,27E+14	olfactory receptor family 5 subfamily E member 1 pseudogene
OR5F1	-3,46E+14	-5,02E+14	2,89E+14	olfactory receptor family 5 subfamily F member 1
OR5H1	-3,23E+14	-4,10E+14	3,79E+14	olfactory receptor family 5 subfamily H member 1
OR5H14	-3,90E+14	-3,21E+14	2,52E+14	olfactory receptor family 5 subfamily H member 14
OR5H15	-3,01E+14	-2,95E+14	5,67E+14	olfactory receptor family 5 subfamily H member 15
OR5H2	-4,24E+14	-2,65E+14	2,93E+14	olfactory receptor family 5 subfamily H member 2
OR5H6	-4,62E+14	-4,62E+14		olfactory receptor family 5 subfamily H member 6 (gene/pseudogene)
OR5I1	-1,53E+14	-3,32E+14		olfactory receptor family 5 subfamily I member 1
OR5J2	-3,11E+14	-4,40E+14	2,19E+12	olfactory receptor family 5 subfamily J member 2
OR5K3	-2,70E+14	-2,36E+14	2,71E+14	olfactory receptor family 5 subfamily K member 3
OR5K4	-3,08E+14	-3,15E+14	2,90E+14	olfactory receptor family 5 subfamily K member 4

OR5L1	-3,02E+14	-3,50E+14	2,76E+14	olfactory receptor family 5 subfamily L member 1 (gene/pseudogene)
OR5L2	-3,86E+14	-3,11E+14	2,76E+14	olfactory receptor family 5 subfamily L member 2
OR5M1	-2,52E+14	-3,38E+14	2,63E+14	olfactory receptor family 5 subfamily M member 1
OR5M10	-4,12E+13	-4,85E+14	2,47E+14	olfactory receptor family 5 subfamily M member 10
OR5M11	-3,18E+14	-3,22E+14	2,46E+14	olfactory receptor family 5 subfamily M member 11
OR5M3	-2,57E+14	-4,83E+14	4,31E+14	olfactory receptor family 5 subfamily M member 3
OR5M8	-4,81E+14	-3,98E+14	3,30E+14	olfactory receptor family 5 subfamily M member 8
OR5M9	-2,26E+14	-2,31E+14	2,51E+14	olfactory receptor family 5 subfamily M member 9
OR5P2	-3,07E+14	-3,52E+14	3,05E+14	olfactory receptor family 5 subfamily P member 2
OR5P3	-3,60E+14	-3,52E+14	2,57E+14	olfactory receptor family 5 subfamily P member 3
OR5R1	-3,16E+14	-2,68E+14	2,70E+14	olfactory receptor family 5 subfamily R member 1 (gene/pseudogene)
OR5T1	-3,21E+14	-2,31E+14	2,91E+14	olfactory receptor family 5 subfamily T member 1
OR5T2	-4,36E+14	-3,03E+14	3,51E+14	olfactory receptor family 5 subfamily T member 2
OR5T3	-3,94E+14	-3,44E+14	3,42E+14	olfactory receptor family 5 subfamily T member 3
OR5V1	-2,67E+14	-2,64E+14	2,34E+14	olfactory receptor family 5 subfamily V member 1
OR5W2	-1,97E+14	-4,09E+14	4,38E+14	olfactory receptor family 5 subfamily W member 2
OR6A2	-3,52E+14	-2,87E+14	2,84E+14	olfactory receptor family 6 subfamily A member 2
OR6B1	-1,97E+14	-3,67E+14	2,28E+13	olfactory receptor family 6 subfamily B member 1
OR6B2	-3,39E+14	-4,35E+14	2,54E+14	olfactory receptor family 6 subfamily B member 2
OR6B3	-2,85E+14	-5,94E+14	3,15E+14	olfactory receptor family 6 subfamily B member 3
OR6C1	-3,22E+14	-3,64E+14	1,82E+14	olfactory receptor family 6 subfamily C member 1
OR6C3	-5,17E+14	-5,17E+14	3,58E+14	olfactory receptor family 6 subfamily C member 3
OR6C4	-7,28E+14	-2,55E+14	3,04E+14	olfactory receptor family 6 subfamily C member 4
OR6C6	-3,16E+13	-3,10E+14	2,51E+14	olfactory receptor family 6 subfamily C member 6
OR6C68	-3,03E+14	-3,41E+13	2,35E+14	olfactory receptor family 6 subfamily C member 68
OR6C70	-4,33E+14	-2,84E+14	2,21E+14	olfactory receptor family 6 subfamily C member 70
OR6C74	-6,13E+14	-3,53E+14	3,06E+14	olfactory receptor family 6 subfamily C member 74
OR6C75	-7,52E+14	-2,37E+14	2,63E+14	olfactory receptor family 6 subfamily C member 75

OR6C76	-3,26E+14	-4,55E+14	3,05E+14	olfactory receptor family 6 subfamily C member 76
OR6F1	-2,04E+14	-2,84E+14	3,48E+14	olfactory receptor family 6 subfamily F member 1
OR6K2	-3,99E+10	-3,03E+14	2,30E+14	olfactory receptor family 6 subfamily K member 2
OR6K3	-1,84E+14	-1,76E+14		olfactory receptor family 6 subfamily K member 3
OR6K6	-1,57E+14	-2,11E+14	2,74E+13	olfactory receptor family 6 subfamily K member 6
OR6M1	-4,49E+14	-3,50E+13	3,86E+14	olfactory receptor family 6 subfamily M member 1
OR6N2	-3,80E+13		1,69E+14	olfactory receptor family 6 subfamily N member 2
OR6P1	-2,00E+14	-3,64E+14	1,93E+14	olfactory receptor family 6 subfamily P member 1
OR6Q1	-3,28E+14	-2,80E+14	3,55E+14	olfactory receptor family 6 subfamily Q member 1 (gene/pseudogene)
OR6S1	-4,18E+14	-3,78E+14	2,19E+14	olfactory receptor family 6 subfamily S member 1
OR6T1	-3,27E+14	-3,17E+14	3,74E+14	olfactory receptor family 6 subfamily T member 1
OR6V1	-3,35E+14	-1,53E+14	2,47E+14	olfactory receptor family 6 subfamily V member 1
OR6W1P	-3,28E+14	-1,53E+14	2,90E+14	olfactory receptor family 6 subfamily W member 1 pseudogene
OR6X1	-3,36E+13	-3,16E+14	2,60E+14	olfactory receptor family 6 subfamily X member 1
OR6Y1	-2,97E+14	-3,27E+14	3,20E+13	olfactory receptor family 6 subfamily Y member 1
OR7A10	-3,88E+14	-4,27E+14	3,06E+14	olfactory receptor family 7 subfamily A member 10
OR7A17	-4,21E+14	-2,71E+14	2,17E+14	olfactory receptor family 7 subfamily A member 17
OR7C1	-2,85E+14	-3,66E+14	1,79E+14	olfactory receptor family 7 subfamily C member 1
OR7C2	-6,77E+14	-2,17E+14	4,46E+14	olfactory receptor family 7 subfamily C member 2 (gene/pseudogene)
OR7D4	-2,71E+14	-3,25E+14	2,12E+14	olfactory receptor family 7 subfamily D member 4
OR7E24	-5,75E+14	-3,15E+14	2,10E+14	olfactory receptor family 7 subfamily E member 24
OR7E37P	-2,06E+14	-1,71E+14	1,48E+13	olfactory receptor family 7 subfamily E member 37 pseudogene
OR7G1	-2,91E+14	-4,04E+13	3,23E+14	olfactory receptor family 7 subfamily G member 1
OR7G2	-3,41E+14	-4,59E+14	2,32E+14	olfactory receptor family 7 subfamily G member 2
OR7G3	-3,65E+14	-3,25E+14	3,01E+13	olfactory receptor family 7 subfamily G member 3
OR8A1	-3,67E+13	-2,65E+13	1,92E+14	olfactory receptor family 8 subfamily A member 1
OR8B12	-2,81E+14	-3,64E+14	3,36E+14	olfactory receptor family 8 subfamily B member 12

OR8B2	-3,29E+14	-3,38E+14	2,70E+14	olfactory receptor family 8 subfamily B member 2
OR8B3	-2,83E+14	-2,82E+14	3,07E+14	olfactory receptor family 8 subfamily B member 3
OR8B4	-3,01E+14	-5,35E+14	2,53E+14	olfactory receptor family 8 subfamily B member 4 (gene/pseudogene)
OR8B8	-3,32E+13	-2,94E+14	2,66E+14	olfactory receptor family 8 subfamily B member 8
OR8D1	-6,78E+14	-4,96E+14	3,66E+14	olfactory receptor family 8 subfamily D member 1
OR8D2	-2,50E+14	-4,06E+14	3,15E+14	olfactory receptor family 8 subfamily D member 2 (gene/pseudogene)
OR8D4	-3,21E+14	-3,89E+13	2,98E+14	olfactory receptor family 8 subfamily D member 4
OR8G1	-2,17E+14	-4,62E+12		olfactory receptor family 8 subfamily G member 1 (gene/pseudogene)
OR8G2P	-4,56E+14	-4,38E+14	2,30E+14	olfactory receptor family 8 subfamily G member 2 pseudogene
OR8G5	-3,07E+14	-1,91E+14	2,38E+14	olfactory receptor family 8 subfamily G member 5
OR8H1	-3,48E+14	-3,53E+14	3,44E+14	olfactory receptor family 8 subfamily H member 1
OR8H2	-2,54E+14	-3,68E+14	3,63E+14	olfactory receptor family 8 subfamily H member 2
OR8H3	-3,66E+14	-3,49E+14	2,97E+13	olfactory receptor family 8 subfamily H member 3
OR8I2	-3,00E+14	-6,33E+14	4,08E+14	olfactory receptor family 8 subfamily I member 2
OR8J1	-3,78E+14	-6,32E+14	3,15E+14	olfactory receptor family 8 subfamily J member 1
OR8J3	-3,06E+14	-2,69E+13	1,63E+14	olfactory receptor family 8 subfamily J member 3
OR8K1	-2,38E+14	-5,72E+13		olfactory receptor family 8 subfamily K member 1
OR8K3	-3,10E+14	-3,51E+14	2,58E+14	olfactory receptor family 8 subfamily K member 3 (gene/pseudogene)
OR8U1	-3,53E+14	-2,61E+14	3,99E+14	olfactory receptor family 8 subfamily U member 1
OR9A4	-3,82E+14	-2,27E+14	1,84E+14	olfactory receptor family 9 subfamily A member 4
OR9G1	-2,78E+13	-4,90E+13	2,65E+14	olfactory receptor family 9 subfamily G member 1
OR9G4	-4,48E+14	-4,42E+14	3,52E+14	olfactory receptor family 9 subfamily G member 4
OR9I1	-3,86E+14	-7,29E+14	2,85E+14	olfactory receptor family 9 subfamily I member 1
OR9K2	-3,15E+14	-3,63E+14	2,57E+14	olfactory receptor family 9 subfamily K member 2
OR9Q2	-4,69E+14	-4,10E+14	2,95E+14	olfactory receptor family 9 subfamily Q member 2
OR1C1			3,97E+14	olfactory receptor family 1 subfamily C member 1

OR1F2P	1,46E+14	olfactory receptor family 1 subfamily F member 2 pseudogene
OR2AK2	1,41E+14	olfactory receptor family 2 subfamily AK member 2
OR2B11	1,83E+14	olfactory receptor family 2 subfamily B member 11
OR2L8	1,70E+14	olfactory receptor family 2 subfamily L member 8 (gene/pseudogene)
OR2T33	1,54E+14	olfactory receptor family 2 subfamily T member 33
OR2T8	2,00E+14	olfactory receptor family 2 subfamily T member 8
OR3A2	2,51E+13	olfactory receptor family 3 subfamily A member 2
OR52W1	2,09E+14	olfactory receptor family 52 subfamily W member 1
OR56A4	2,78E+14	olfactory receptor family 56 subfamily A member 4
OR5A1	2,13E+14	olfactory receptor family 5 subfamily A member 1
OR7E156P	4,71E+13	olfactory receptor family 7 subfamily E member 156 pseudogene
OR9A2	2,63E+14	olfactory receptor family 9 subfamily A member 2

Supplementary Table S2. Olfactory Receptors (ORs) differentially expressed included and used in hierarchical clustering. We show the logarithmic fold change (logFC) of the 93 ORs used to construct the hierarchical clustering from mild nontreated gestational hypothyroidism (GHT) compared with the healthy thyroid pregnancy (HTP). We also show the ORs differentially expressed in preeclampsia (PEC) and HTP. Healthy nonpregnant women (NPG) libraries were used to evaluate the DEG in HTP.

Symbol	LogFC.GHT_HTP	LogFC.PEC_HTP	LogFC.HTP_NPG	Name
OR10A3	-4,32E+14	-3,58E+14	2,90E+14	olfactory receptor family 10 subfamily A member 3
OR10A7	-3,57E+14	-3,13E+14	3,09E+14	olfactory receptor family 10 subfamily A member 7
OR10C1	-3,56E+14	-3,60E+11	3,20E+14	olfactory receptor family 10 subfamily C member 1 (gene/pseudogene)
OR10G3	-3,44E+14	-3,83E+14	2,00E+14	olfactory receptor family 10 subfamily G member 3
OR10G4	-3,18E+14	-3,53E+14	2,96E+14	olfactory receptor family 10 subfamily G member 4
OR10G7	-3,51E+14	-4,45E+13	2,37E+14	olfactory receptor family 10 subfamily G member 7
OR10G8	-3,61E+14	-3,26E+14	3,15E+14	olfactory receptor family 10 subfamily G member 8
OR10H4	-3,88E+14	-3,81E+14	2,68E+14	olfactory receptor family 10 subfamily H member 4
OR10J5	-3,97E+14	-3,62E+14	2,68E+14	olfactory receptor family 10 subfamily J member 5
OR11H12	-3,93E+14	-4,02E+14	3,25E+14	olfactory receptor family 11 subfamily H member 12
OR12D2	-3,80E+14	-3,36E+14	2,29E+14	olfactory receptor family 12 subfamily D member 2 (gene/pseudogene)
OR12D3	-4,48E+14	-5,25E+14	3,01E+14	olfactory receptor family 12 subfamily D member 3
OR13C2	-3,72E+14	-4,29E+14	2,54E+14	olfactory receptor family 13 subfamily C member 2
OR13F1	-3,12E+14	-3,53E+14	2,43E+14	olfactory receptor family 13 subfamily F member 1
OR1J2	-3,77E+14	-3,67E+14	2,26E+14	olfactory receptor family 1 subfamily J member 2
OR1J4	-3,70E+14	-3,89E+14	1,92E+14	olfactory receptor family 1 subfamily J member 4
OR1S2	-3,44E+14	-4,25E+14	3,55E+14	olfactory receptor family 1 subfamily S member 2
OR2A14	-4,00E+14	-3,34E+14	2,00E+14	olfactory receptor family 2 subfamily A member 14
OR2AG1	-3,60E+14	-3,19E+14	2,19E+14	olfactory receptor family 2 subfamily AG member 1 (gene/pseudogene)
OR2AG2	-4,23E+14	-4,15E+14	2,35E+14	olfactory receptor family 2 subfamily AG member 2
OR2F2	-4,11E+14	-3,48E+14	1,65E+14	olfactory receptor family 2 subfamily F member 2
OR2G2	-3,02E+14	-3,79E+14	2,17E+13	olfactory receptor family 2 subfamily G member 2
OR2G6	-3,69E+14	-3,36E+14	1,82E+14	olfactory receptor family 2 subfamily G member 6
OR2K2	-3,44E+14	-4,37E+13	2,77E+14	olfactory receptor family 2 subfamily K member 2

OR2T1	-3,32E+14	-3,51E+14	2,90E+14	olfactory receptor family 2 subfamily T member 1
OR2T27	-3,67E+14	-3,76E+13	3,20E+14	olfactory receptor family 2 subfamily T member 27
OR2T4	-3,51E+14	-4,06E+14	3,15E+14	olfactory receptor family 2 subfamily T member 4
OR2V2	-3,71E+14	-3,17E+14	2,58E+14	olfactory receptor family 2 subfamily V member 2
OR2Z1	-3,41E+14	-3,31E+14	2,12E+14	olfactory receptor family 2 subfamily Z member 1
OR4A15	-3,06E+14	-3,44E+14	1,90E+14	olfactory receptor family 4 subfamily A member 15
OR4A5	-3,33E+14	-4,09E+14	4,53E+14	olfactory receptor family 4 subfamily A member 5
OR4B1	-3,36E+14	-3,62E+14	2,53E+14	olfactory receptor family 4 subfamily B member 1
OR4C12	-6,28E+14	-6,28E+14	2,35E+14	olfactory receptor family 4 subfamily C member 12
OR4C15	-4,69E+14	-5,41E+14	4,46E+14	olfactory receptor family 4 subfamily C member 15
OR4C3	-4,17E+14	-3,62E+14	2,07E+14	olfactory receptor family 4 subfamily C member 3
OR4C46	-3,18E+14	-3,39E+13	3,00E+14	olfactory receptor family 4 subfamily C member 46
OR4C6	-3,74E+14	-3,12E+14	3,55E+14	olfactory receptor family 4 subfamily C member 6
OR4D2	-3,20E+14	-3,08E+14	1,88E+14	olfactory receptor family 4 subfamily D member 2
OR4D9	-3,79E+14	-3,20E+14	2,69E+14	olfactory receptor family 4 subfamily D member 9
OR4E2	-3,44E+14	-3,05E+13	2,64E+14	olfactory receptor family 4 subfamily E member 2 (gene/pseudogene)
OR4K15	-4,23E+14	-3,35E+14	2,97E+14	olfactory receptor family 4 subfamily K member 15
OR4K5	-3,07E+13	-3,00E+14	2,88E+14	olfactory receptor family 4 subfamily K member 5
OR4L1	-3,52E+13	-3,17E+14	4,05E+14	olfactory receptor family 4 subfamily L member 1 (gene/pseudogene)
OR4M1	-4,59E+14	-4,89E+14	3,37E+14	olfactory receptor family 4 subfamily M member 1
OR4M2	-3,89E+14	-4,34E+14	3,63E+14	olfactory receptor family 4 subfamily M member 2
OR4N3P	-4,21E+14	-4,30E+14	2,92E+14	olfactory receptor family 4 subfamily N member 3 pseudogene
OR4N5	-3,85E+14	-3,47E+14	2,86E+14	olfactory receptor family 4 subfamily N member 5
OR4X1	-3,46E+14	-3,60E+14	3,51E+14	olfactory receptor family 4 subfamily X member 1 (gene/pseudogene)
OR51A7	-3,87E+14	-3,88E+14	3,51E+14	olfactory receptor family 51 subfamily A member 7
OR51B4	-3,77E+14	-3,22E+14	2,85E+14	olfactory receptor family 51 subfamily B member 4
OR51B6	-3,59E+14	-3,42E+14	2,57E+14	olfactory receptor family 51 subfamily B member 6

OR51G2	-5,10E+14	-4,98E+13	3,04E+13	olfactory receptor family 51 subfamily G member 2
OR51V1	-3,07E+13	-3,97E+14	3,02E+14	olfactory receptor family 51 subfamily V member 1
OR52E2	-3,63E+14	-3,09E+14	2,74E+14	olfactory receptor family 52 subfamily E member 2
OR52E8	-4,82E+13	-3,87E+14	2,91E+13	olfactory receptor family 52 subfamily E member 8
OR56A3	-3,60E+14	-3,65E+14	2,54E+14	olfactory receptor family 56 subfamily A member 3
OR56A5	-3,33E+14	-3,25E+14	2,45E+14	olfactory receptor family 56 subfamily A member 5
OR5AK4P	-4,39E+14	-4,27E+14	3,31E+14	olfactory receptor family 5 subfamily AK member 4 pseudogene
OR5B12	-3,12E+13	-3,42E+13	3,24E+13	olfactory receptor family 5 subfamily B member 12
OR5E1P	-5,99E+14	-5,99E+14	2,27E+14	olfactory receptor family 5 subfamily E member 1 pseudogene
OR5H1	-3,23E+14	-4,10E+14	3,79E+14	olfactory receptor family 5 subfamily H member 1
OR5H14	-3,90E+14	-3,21E+14	2,52E+14	olfactory receptor family 5 subfamily H member 14
OR5H6	-4,62E+14	-4,62E+14		olfactory receptor family 5 subfamily H member 6 (gene/pseudogene)
OR5K4	-3,08E+14	-3,15E+14	2,90E+14	olfactory receptor family 5 subfamily K member 4
OR5L1	-3,02E+14	-3,50E+14	2,76E+14	olfactory receptor family 5 subfamily L member 1 (gene/pseudogene)
OR5L2	-3,86E+14	-3,11E+14	2,76E+14	olfactory receptor family 5 subfamily L member 2
OR5M10	-4,12E+13	-4,85E+14	2,47E+14	olfactory receptor family 5 subfamily M member 10
OR5M11	-3,18E+14	-3,22E+14	2,46E+14	olfactory receptor family 5 subfamily M member 11
OR5M8	-4,81E+14	-3,98E+14	3,30E+14	olfactory receptor family 5 subfamily M member 8
OR5P2	-3,07E+14	-3,52E+14	3,05E+14	olfactory receptor family 5 subfamily P member 2
OR5P3	-3,60E+14	-3,52E+14	2,57E+14	olfactory receptor family 5 subfamily P member 3
OR5T3	-3,94E+14	-3,44E+14	3,42E+14	olfactory receptor family 5 subfamily T member 3
OR6B2	-3,39E+14	-4,35E+14	2,54E+14	olfactory receptor family 6 subfamily B member 2
OR6C1	-3,22E+14	-3,64E+14	1,82E+14	olfactory receptor family 6 subfamily C member 1
OR6C3	-5,17E+14	-5,17E+14	3,58E+14	olfactory receptor family 6 subfamily C member 3
OR6C6	-3,16E+13	-3,10E+14	2,51E+14	olfactory receptor family 6 subfamily C member 6
OR6C68	-3,03E+14	-3,41E+13	2,35E+14	olfactory receptor family 6 subfamily C member 68
OR6K2	-3,99E+10	-3,03E+14	2,30E+14	olfactory receptor family 6 subfamily K member 2

OR6M1	-4,49E+14	-3,50E+13	3,86E+14	olfactory receptor family 6 subfamily M member 1
OR6S1	-4,18E+14	-3,78E+14	2,19E+14	olfactory receptor family 6 subfamily S member 1
OR6T1	-3,27E+14	-3,17E+14	3,74E+14	olfactory receptor family 6 subfamily T member 1
OR6X1	-3,36E+13	-3,16E+14	2,60E+14	olfactory receptor family 6 subfamily X member 1
OR7A10	-3,88E+14	-4,27E+14	3,06E+14	olfactory receptor family 7 subfamily A member 10
OR7G3	-3,65E+14	-3,25E+14	3,01E+13	olfactory receptor family 7 subfamily G member 3
OR8B2	-3,29E+14	-3,38E+14	2,70E+14	olfactory receptor family 8 subfamily B member 2
OR8D4	-3,21E+14	-3,89E+13	2,98E+14	olfactory receptor family 8 subfamily D member 4
OR8G2P	-4,56E+14	-4,38E+14	2,30E+14	olfactory receptor family 8 subfamily G member 2 pseudogene
OR8H1	-3,48E+14	-3,53E+14	3,44E+14	olfactory receptor family 8 subfamily H member 1
OR8H3	-3,66E+14	-3,49E+14	2,97E+13	olfactory receptor family 8 subfamily H member 3
OR8K3	-3,10E+14	-3,51E+14	2,58E+14	olfactory receptor family 8 subfamily K member 3 (gene/pseudogene)
OR9G4	-4,48E+14	-4,42E+14	3,52E+14	olfactory receptor family 9 subfamily G member 4
OR9K2	-3,15E+14	-3,63E+14	2,57E+14	olfactory receptor family 9 subfamily K member 2
OR9Q2	-4,69E+14	-4,10E+14	2,95E+14	olfactory receptor family 9 subfamily Q member 2

Supplementary Table S3. Olfactory Receptors (ORs) differentially expressed excluded. We removed 102 ORs that presented logarithmic fold change (logFC) cutoffs of > – 3.0 at least in one dataset (mild

nontreated gestational hypothyroidism - GHT, preeclampsia - PEC and healthy thyroid pregnancy - HTP). The table presented the LogFC from each group. Healthy nonpregnant women (NPG) was used to call the HTP DEG.

Symbol	LogFC.GHT_HTP	LogFC.PEC_HTP	LogFC.HTP_NPG	Name
OR10A2	-3,22E+13	-2,78E+14	2,63E+14	olfactory receptor family 10 subfamily A member 2
OR10A5	-3,30E+14	-2,49E+14	2,30E+14	olfactory receptor family 10 subfamily A member 5
OR10H5	-2,56E+14	-3,26E+14	2,46E+12	olfactory receptor family 10 subfamily H member 5
OR10J3	-2,32E+14	-2,75E+14	2,42E+14	olfactory receptor family 10 subfamily J member 3
OR10K1	-2,68E+14	-2,76E+14	2,91E+14	olfactory receptor family 10 subfamily K member 1
OR10K2	-2,81E+14	-2,71E+14	2,26E+14	olfactory receptor family 10 subfamily K member 2
OR10V1	-2,45E+14	-2,67E+14	2,80E+14	olfactory receptor family 10 subfamily V member 1
OR10W1	-2,82E+14	-3,08E+14	2,58E+14	olfactory receptor family 10 subfamily W member 1
OR10Z1	-2,25E+14	-2,43E+14	2,50E+13	olfactory receptor family 10 subfamily Z member 1
OR11G2	-3,23E+14	-2,53E+14	3,02E+14	olfactory receptor family 11 subfamily G member 2
OR11H1	-2,82E+13	-3,11E+14	3,13E+14	olfactory receptor family 11 subfamily H member 1
OR11H2	-3,09E+14	-2,76E+14	3,47E+14	olfactory receptor family 11 subfamily H member 2
OR13C8	-3,63E+14	-2,94E+14	2,04E+14	olfactory receptor family 13 subfamily C member 8
OR13J1	-2,47E+14	-3,33E+14	2,22E+14	olfactory receptor family 13 subfamily J member 1
OR14A16	-2,89E+14	-2,97E+14	2,72E+14	olfactory receptor family 14 subfamily A member 16
OR14J1	-2,31E+14	-2,77E+14	3,07E+14	olfactory receptor family 14 subfamily J member 1
OR1A2	-3,48E+14	-2,53E+14	3,48E+14	olfactory receptor family 1 subfamily A member 2
OR1D5	-2,86E+14	-3,24E+13	2,03E+14	olfactory receptor family 1 subfamily D member 5
OR1E1	-2,65E+14	-3,47E+14	3,08E+14	olfactory receptor family 1 subfamily E member 1
OR1E2	-3,12E+14	-2,90E+14	2,67E+12	olfactory receptor family 1 subfamily E member 2
OR1F1	-3,50E+14	-2,76E+14	1,96E+13	olfactory receptor family 1 subfamily F member 1
OR1J1	-3,01E+14	-2,99E+14	2,00E+13	olfactory receptor family 1 subfamily J member 1
OR1L1	-2,90E+14	-2,77E+14	2,31E+14	olfactory receptor family 1 subfamily L member 1
OR1L4	-1,93E+14	-1,46E+14	1,75E+14	olfactory receptor family 1 subfamily L member 4
OR1L6	-2,03E+14	-1,99E+14		olfactory receptor family 1 subfamily L member 6
OR1N2	-3,24E+14	-2,41E+14	2,22E+14	olfactory receptor family 1 subfamily N member 2

OR1S1	-2,60E+14	-2,77E+14	2,99E+14	olfactory receptor family 1 subfamily S member 1 (gene/pseudogene)
OR2A2	-3,43E+14	-2,84E+14	3,25E+14	olfactory receptor family 2 subfamily A member 2
OR2A25	-3,33E+14	-2,34E+14	2,72E+14	olfactory receptor family 2 subfamily A member 25
OR2B2	-2,11E+14	-2,75E+14	1,78E+14	olfactory receptor family 2 subfamily B member 2
OR2D3	-2,75E+14	-2,83E+14	2,12E+13	olfactory receptor family 2 subfamily D member 3
OR2H2	-2,29E+14	-2,05E+14	1,96E+14	olfactory receptor family 2 subfamily H member 2
OR2J3	-3,18E+14	-2,64E+14	2,43E+14	olfactory receptor family 2 subfamily J member 3
OR2L3	-2,94E+14	-2,84E+14	2,47E+14	olfactory receptor family 2 subfamily L member 3
OR2M2	-2,13E+14	-2,57E+13	1,84E+14	olfactory receptor family 2 subfamily M member 2
OR2M3	-3,28E+14	-2,36E+14		olfactory receptor family 2 subfamily M member 3
OR2M4	-2,45E+14	-3,08E+14	3,87E+14	olfactory receptor family 2 subfamily M member 4
OR2T10	-2,54E+14	-2,56E+14	1,58E+14	olfactory receptor family 2 subfamily T member 10
OR2T2	-2,50E+13	-2,93E+14	2,40E+14	olfactory receptor family 2 subfamily T member 2
OR2T29	-2,87E+14	-2,46E+14	3,45E+14	olfactory receptor family 2 subfamily T member 29
OR2T35	-2,27E+14	-1,94E+13	1,88E+14	olfactory receptor family 2 subfamily T member 35
OR2T6	-2,82E+14	-3,64E+14	1,91E+14	olfactory receptor family 2 subfamily T member 6
OR2Y1	-2,61E+14	-2,65E+14	1,60E+14	olfactory receptor family 2 subfamily Y member 1
OR3A1	-3,17E+14	-2,43E+14	1,57E+14	olfactory receptor family 3 subfamily A member 1 (gene/pseudogene)
OR3A4P	-2,73E+14	-3,60E+14	3,09E+14	olfactory receptor family 3 subfamily A member 4 pseudogene
OR4A16	-2,69E+14	-3,21E+14	2,58E+14	olfactory receptor family 4 subfamily A member 16
OR4A47	-2,68E+14	-2,85E+14	2,36E+14	olfactory receptor family 4 subfamily A member 47
OR4C13	-2,71E+14	-3,01E+14	3,65E+14	olfactory receptor family 4 subfamily C member 13
OR4D10	-3,29E+14	-2,81E+14	1,96E+14	olfactory receptor family 4 subfamily D member 10
OR4D5	-2,89E+14	-3,27E+14	2,51E+13	olfactory receptor family 4 subfamily D member 5
OR4F15	-3,11E+14	-2,77E+14	1,65E+14	olfactory receptor family 4 subfamily F member 15
OR4F21	-2,14E+14	-1,27E+14	1,83E+14	olfactory receptor family 4 subfamily F member 21
OR4F4	-2,74E+14	-1,91E+14	3,42E+14	olfactory receptor family 4 subfamily F member 4
OR4N4	-2,85E+14	-2,50E+14	2,36E+14	olfactory receptor family 4 subfamily N member 4

OR4X2	-2,91E+14	-2,65E+14	3,46E+14	olfactory receptor family 4 subfamily X member 2 (gene/pseudogene)
OR51A4	-2,77E+14	-3,34E+14	2,69E+13	olfactory receptor family 51 subfamily A member 4
OR51B2	-2,47E+14	-2,24E+14	3,13E+14	olfactory receptor family 51 subfamily B member 2 (gene/pseudogene)
OR51G1	-2,51E+14	-2,35E+14	1,84E+14	olfactory receptor family 51 subfamily G member 1 (gene/pseudogene)
OR51I1	-2,34E+14	-3,18E+14	3,05E+14	olfactory receptor family 51 subfamily I member 1
OR51L1	-2,86E+13	-3,16E+14	2,79E+14	olfactory receptor family 51 subfamily L member 1
OR51M1	-2,28E+14	-2,35E+14	2,01E+14	olfactory receptor family 51 subfamily M member 1
OR51Q1	-3,44E+14	-2,57E+14	2,17E+14	olfactory receptor family 51 subfamily Q member 1 (gene/pseudogene)
OR51T1	-2,57E+14	-3,27E+14	2,96E+14	olfactory receptor family 51 subfamily T member 1
OR52A5	-2,71E+14	-3,59E+14	3,08E+14	olfactory receptor family 52 subfamily A member 5
OR52B4	-2,67E+11	-2,74E+14	3,06E+14	olfactory receptor family 52 subfamily B member 4 (gene/pseudogene)
OR52D1	-2,89E+14	-3,74E+14	2,67E+14	olfactory receptor family 52 subfamily D member 1 (gene/pseudogene)
OR52E4	-2,48E+14	-2,87E+14	4,85E+14	olfactory receptor family 52 subfamily E member 4
OR52I2	-1,75E+14	-2,64E+14	1,56E+14	olfactory receptor family 52 subfamily I member 2
OR52J3	-2,89E+14	-3,19E+14	2,51E+14	olfactory receptor family 52 subfamily J member 3
OR52L1	-2,24E+14	-2,11E+14	2,40E+14	olfactory receptor family 52 subfamily L member 1 (gene/pseudogene)
OR52N5	-2,73E+14	-2,59E+14	2,12E+14	olfactory receptor family 52 subfamily N member 5
OR52R1	-3,45E+14	-2,84E+13	3,14E+14	olfactory receptor family 52 subfamily R member 1 (gene/pseudogene)
OR56B4	-2,78E+14	-1,79E+14	2,49E+14	olfactory receptor family 56 subfamily B member 4
OR5AN1	-2,83E+14	-2,87E+14	2,96E+14	olfactory receptor family 5 subfamily AN member 1
OR5AS1	-2,80E+14	-1,84E+13	2,05E+14	olfactory receptor family 5 subfamily AS member 1
OR5AU1	-2,70E+14	-3,41E+13	2,88E+14	olfactory receptor family 5 subfamily AU member 1
OR5B2	-3,28E+11	-2,36E+14	2,49E+14	olfactory receptor family 5 subfamily B member 2
OR5B3	-2,42E+14	-3,24E+14		olfactory receptor family 5 subfamily B member 3
OR5C1	-2,73E+14	-1,81E+14	1,83E+14	olfactory receptor family 5 subfamily C member 1

OR5D13	-2,49E+14	-3,03E+14	1,93E+14	olfactory receptor family 5 subfamily D member 13 (gene/pseudogene)
OR5D14	-3,57E+14	-2,83E+14	2,84E+14	olfactory receptor family 5 subfamily D member 14
OR5H15	-3,01E+14	-2,95E+14	5,67E+14	olfactory receptor family 5 subfamily H member 15
OR5K3	-2,70E+14	-2,36E+14	2,71E+14	olfactory receptor family 5 subfamily K member 3
OR5M1	-2,52E+14	-3,38E+14	2,63E+14	olfactory receptor family 5 subfamily M member 1
OR5M9	-2,26E+14	-2,31E+14	2,51E+14	olfactory receptor family 5 subfamily M member 9
OR5R1	-3,16E+14	-2,68E+14	2,70E+14	olfactory receptor family 5 subfamily R member 1 (gene/pseudogene)
OR5T1	-3,21E+14	-2,31E+14	2,91E+14	olfactory receptor family 5 subfamily T member 1
OR5V1	-2,67E+14	-2,64E+14	2,34E+14	olfactory receptor family 5 subfamily V member 1
OR6A2	-3,52E+14	-2,87E+14	2,84E+14	olfactory receptor family 6 subfamily A member 2
OR6F1	-2,04E+14	-2,84E+14	3,48E+14	olfactory receptor family 6 subfamily F member 1
OR6K3	-1,84E+14	-1,76E+14		olfactory receptor family 6 subfamily K member 3
OR6K6	-1,57E+14	-2,11E+14	2,74E+13	olfactory receptor family 6 subfamily K member 6
OR6Q1	-3,28E+14	-2,80E+14	3,55E+14	olfactory receptor family 6 subfamily Q member 1 (gene/pseudogene)
OR6Y1	-2,97E+14	-3,27E+14	3,20E+13	olfactory receptor family 6 subfamily Y member 1
OR7C1	-2,85E+14	-3,66E+14	1,79E+14	olfactory receptor family 7 subfamily C member 1
OR7D4	-2,71E+14	-3,25E+14	2,12E+14	olfactory receptor family 7 subfamily D member 4
OR7E37P	-2,06E+14	-1,71E+14	1,48E+13	olfactory receptor family 7 subfamily E member 37 pseudogene
OR8B12	-2,81E+14	-3,64E+14	3,36E+14	olfactory receptor family 8 subfamily B member 12
OR8B3	-2,83E+14	-2,82E+14	3,07E+14	olfactory receptor family 8 subfamily B member 3
OR8B8	-3,32E+13	-2,94E+14	2,66E+14	olfactory receptor family 8 subfamily B member 8
OR8J3	-3,06E+14	-2,69E+13	1,63E+14	olfactory receptor family 8 subfamily J member 3
OR8U1	-3,53E+14	-2,61E+14	3,99E+14	olfactory receptor family 8 subfamily U member 1
